# Spatial Distribution of Cerebral White Matter Lesions Predicts Progression to Mild Cognitive Impairment and Dementia

**DOI:** 10.1371/journal.pone.0056972

**Published:** 2013-02-14

**Authors:** Marion Mortamais, Christelle Reynes, Adam M. Brickman, Frank A. Provenzano, Jordan Muraskin, Florence Portet, Claudine Berr, Jacques Touchon, Alain Bonafé, Emmanuelle le Bars, Jerome J. Maller, Chantal Meslin, Robert Sabatier, Karen Ritchie, Sylvaine Artero

**Affiliations:** 1 Inserm, U1061, La Colombière Hospital, Montpellier, France; 2 University of Montpellier 1, Montpellier, France; 3 EA 2415, Faculté de Pharmacie, Montpellier, France; 4 Taub Institute for Research on Alzheimer's Disease and the Aging Brain, Columbia University, College of Physicians and Surgeons, New York, New York, United States of America; 5 Unité Transversale des Troubles Neurologiques du Sujet Âgé, CHU Caremeau, Centre Ruffi, Pôle de Gériatrie, CHU Nîmes, Nîmes, France; 6 Montpellier University Hospital, University Department of Adult Psychiatry, La Colombière Hospital, CHU de Montpellier, Montpellier, France; 7 CHRU Montpellier, Montpellier, France; 8 Monash Alfred Psychiatry Research Centre, The Alfred and Monash University Central Clinical School, Melbourne, Australia; 9 Centre for Mental Health Research, Australian National University, Canberra, Australia; 10 Faculty of Medicine, Imperial College, London, United Kingdom; McGill University/Douglas Mental Health Univ. Institute, Canada

## Abstract

**Context:**

White matter lesions (WML) increase the risk of dementia. The relevance of WML location is less clear. We sought to determine whether a particular WML profile, based on the density and location of lesions, could be associated with an increased risk of mild cognitive impairment (MCI) or dementia over the following 7 years.

**Methods:**

In 426 healthy subjects from a cohort of community-dwelling people aged 65 years and over (ESPRIT Project), standardized cognitive and neurological evaluations were repeated after 2, 4 and 7 years. Patterns of WML were computed with a supervised data mining approach (decision trees) using the regional WML volumes (frontal, parietal, temporal, and occipital regions) and the total WML volume estimated at baseline. Cox proportional hazard models were then constructed to study the association between WML patterns and risk of MCI/dementia.

**Results:**

Total WML volume and percentage of WML in the temporal region proved to be the best predictors of progression to MCI and dementia. Specifically, severe total WML load with a high proportion of lesions in the temporal region was significantly associated with the risk of developing MCI or dementia.

**Conclusions:**

Above a certain threshold of damage, a pattern of WML clustering in the temporal region identifies individuals at increased risk of MCI or dementia. As this WML pattern is observed before the onset of clinical symptoms, it may facilitate the detection of patients at risk of MCI/dementia.

## Introduction

Cerebral white matter lesions (WML) are commonly found on magnetic resonance imaging (MRI) scans of elderly people. WML are thought to be the result of degenerative changes in small vessels [1], and hypertension and arteriosclerosis are considered significant risk factors [2], [3]. Although several studies indicate that WML load is associated with cognitive decline and incident dementia [4], other works do not support such relationship [5], [6]. The association of WML with cognitive impairment in normal aging and dementia is still not fully understood. The regional distribution of WML might help understanding the relationship between WML with cognitive impairment and dementia, because not only the extent, but also the localization of WML could determine its clinical significance. A recent study showed that specific WML loci were closely associated with poor executive function and episodic memory, independently of the total WML load [7]. WML may cause cognitive impairment by disrupting cortical connections that are mediated by specific white matter (WM) tracts. Diffusion tensor imaging (DTI) and volumetric MRI studies have demonstrated that WM degenerates from the anterior to the posterior part of the brain in normal aging [8], [9]. Conflicting results have been reported concerning the extent and spatial distribution of WM changes in patients with Mild Cognitive Impairment (MCI) or dementia. While some studies did not find a particular regional distribution of WM changes in Alzheimer's disease (AD) [10], [11], [12], others revealed significant higher WM deterioration in posterior regions in AD [13], [14] and also in MCI, albeit to a lesser extent. As in normal aging WM deterioration shows antero-posterior progression [8], the association between WM deterioration in the posterior regions with the risk of MCI or dementia suggests a continuum from normal aging to dementia [12]. The respective influence of global and localized WM changes is difficult to distinguish. Previous research used a “top-down approach” to compare WML frequency in healthy and dementia/MCI groups, which were defined using criteria that do not take into account WML. In the present study, we employed an alternative and original “bottom-up” approach, using a supervised data mining method (decision trees) to investigate whether and to what extent the regional and total WML volumes could specifically discriminate healthy from cognitively impaired individuals within a single study group. Our aim was to determine whether in a large cohort of cognitively healthy elderly people from a longitudinal population-based study, specific WML patterns (density and location) could be identified that may ultimately be linked to the risk of MCI or dementia over a 7-year period.

## Methods

### Study population

Between 1999 and 2001, 1863 people aged 65 years and over were recruited from the electoral rolls for the ESPRIT Project (Montpellier, France). The study design was described elsewhere [15]. The study protocol was approved by the National Ethical Committee and written informed consent was obtained from each participant. Examinations comprised a standardized interview, neuropsychological tests and a standardized neurological examination at baseline and after 2, 4 and 7 years. At baseline, 760 participants under the age of 80 were randomly selected and invited to have an MRI brain scan. For the present study, we excluded 43 people who did not have MRI images of sufficiently good quality to quantify the total WML volume and 204 participants with missing data for covariates (regional WML volume: n = 174; total brain volume: n = 20; Apolipoprotein E4 (APOE 4) genotype: n = 5; depressive symptomatology: n = 5). This group was further reduced by eliminating subjects who received a diagnosis of dementia (n = 10) or MCI (n = 77) at baseline. Of the remaining 426 participants with an MRI brain scan, 55% were women and the median age was 71 years for men and 70 for women.

### MRI imaging

#### Estimation of brain volume, brain atrophy, hippocampal volume and silent brain infarcts

A 1.5T MRI scanner (Signa Advantage Echospeed; GE Healthcare, Milwaukee, Wis) was used to acquire anterior commissure-posterior commissure aligned fast SPGR 3D T1-weighted anatomical images of 1mm thickness which were then segmented with SPM 5 (Wellcome Department of Cognitive Neurology, [http://www.fil.ion.ucl.ac.uk/spm/software/spm5/]) to derive grey matter and WM volumes. The total brain volume was then calculated as the sum of volumes of grey matter and WM and used as a covariate to adjust for brain size. Brain atrophy was determined as the percentage of the volume of cerebrospinal fluid (CSF) relative to the intracranial volume (sum of grey matter, WM and CSF volumes). Hippocampal regions of interest (ROIs) were manually outlined on consecutive coronal slices and the axial and sagittal orientations verified [16]. The complete method was described elsewhere [17]. Hippocampal volume could be estimated in 424 participants; 2 participants had images of insufficient quality to quantify this volume.

Presence of silent brain infarcts (SBI) was assessed visually by a neurologist (FP) in 418 participants after exclusion of patients with stroke (n = 8). SBI were defined as focal hyper-intensity areas (≥3 mm in size) on T2-weighted images, measured using dedicated software (Myrian®, Intrasense).

#### Estimation of WML volume

For each participant, MRI structural imaging was performed using fast multislice double echo T2-weighted 2D axial acquisition, 4 mm thick slices, with 0.4 mm between-slice spacing that covered the whole brain (30 slices, the upper slice passing through the brain vertex). WML volume was estimated using a semi-automatic method [18], [19]. Areas of supratentorial WML that appeared as hyperintensity zones were segmented on T2 sequences using the MRIcro software [20]. WML in basal ganglia and the infratentorial region were not assessed for the present study. A first layer of ROIs corresponding to WML was created by using a semi-automated technique based on intensity thresholding. A second layer of ROIs was then manually outlined on each slide by roughly contouring all WML. The intersection between the first and the second layer of ROIs was then manually inspected and automatic total WML volume obtained. An experienced reader blind to the follow-up outcome (EB) examined all scans. A neurologist (FP) examined 80 randomly chosen scans to assess the inter-rater reliability. The inter-reader and intra-reader, intra-class correlation coefficients showed good to excellent agreement (0.79 and 0.95 respectively).

The localization of WML in the brain was assessed quantitatively [21]; T1-weighted SPGR sequences were spatially normalized into standard atlas space and the inverse transformation was interpreted using a WM atlas [22] to define WML in the frontal, parietal, occipital and temporal regions. Finally, the total WML volume was calculated by adding up the WML volumes measured in the four regions. We also constructed variables representing the relative WML volume (WMLr) in the different regions by dividing the regional WML volume by the total WML volume (specific location/total WML volume) ×100 to obtain the frontal WMLr, parietal WMLr, temporal WMLr and occipital WMLr.

### Diagnosis of dementia and MCI

Preliminary diagnoses of dementia made by a neurologist according to the DSM IV criteria [23] at baseline and at each follow-up examination were validated by an independent national panel of neurologists to obtain consensus. Although the type of dementia was determined during the clinical interview, we did not distinguish between AD and other types of dementia in our analysis due to the low number of incident cases. Specifically, among the 11 participants who developed dementia, AD was diagnosed in 8 subjects (73%), vascular dementia in 1 (9%) and other forms of dementia in 2 (18%). MCI (which is considered a prodrome to dementia) was diagnosed according to the currently used, revised criteria for MCI (MCI-R algorithm), proposed by an international consensus group [24]. We previously showed that the MCI-R algorithm allows a better prediction of the cognitive deficits that will progress towards dementia than the original MCI criteria [25]. Briefly, MCI was diagnosed in the presence of a cognitive complaint and a score within the 20^th^ percentile for the relevant age-matched and education-matched group in at least one of the tests from the short cognitive battery included in the study (Benton Visual Retention Test [26], Isaacs' Set Test of verbal fluency [27] and immediate and delayed recall of the 5 Word-Test of Dubois [28]). We used percentiles rather than standard deviations, given the non-normal distribution of the cognitive scores. Study participants with incident MCI are at high risk of developing dementia after the 7 years of follow-up and therefore cannot be classified as normal subjects.

We chose to group together patients with dementia or MCI. Separate dementia and MCI groups may be preferable for analytical studies that sought to clarify causation; however, our main objective was to identify baseline WML patterns that could help discriminating study participants who were at risk of progressing towards dementia and its pre-clinical prodrome. The date of onset of dementia or MCI was set half way between the date of the last follow-up visit when the subject was classified as normal and the date of diagnosis.

### Socio-demographic and clinical factors

The standardized interview included questions on socio-demographic characteristics and education level (no formal education, primary, secondary and higher education). Depressive symptomatology was assessed with the Center for Epidemiologic Studies-Depression Scale (CESD) [29] with a >16 cut-off score indicating a high level of symptomatology. Blood pressure was measured with a digital OMRON M4 blood pressure monitor twice during the interview. Subjects were considered hypertensive when the mean of the two measures was ≥160/90 mm Hg or if they were taking anti-hypertensive drugs. History of stroke was self-reported. Vascular pathology antecedents included history of stroke, angina pectoris, myocardial infarction, coronary surgery, coronary angioplasty and arterial surgery of the legs for arteritis. APOE 4 genotype (presence or absence of the allele ε4) (http://www.genopole-lille.fr/spip/) was also included in the statistical analyses.

### Statistical analysis

#### Construction of WML patterns

We used decision trees [30] to profile subjects who developed MCI/dementia in an attempt to define specific WML patterns that might be associated with increased risk of MCI/dementia. A decision tree is a machine-learning method for building models that can be easily understood and interpreted. It successively uses automatically selected descriptors through binary questions to partition the subjects into subsets that are as homogeneous as possible for the response variable value (which is to be predicted).

Decision trees were chosen here because they provide completely “white-box” multivariate models while performing automated feature selection. The final model can be directly used to understand how the decision is made. Moreover, the use of threshold values is of major utility in the clinical context. Finally, the model simplicity combined with the used cross-validation process makes the method less prone to overfitting. In our study, decision trees were built to determine which descriptors (among total WML volume, frontal WMLr, parietal WMLr, temporal WMLr and occipital WMLr) could discriminate between subjects who developed MCI/dementia over a 7 year period and subjects who remained cognitively healthy. In this context, a decision tree classified all subjects (healthy or with incident MCI/dementia) by successively posing a series of binary questions about the value of one WML feature (i.e., “is the value of a given WML volume higher than a given threshold value or not?”). Each question is contained in a node, and at each node, decision trees recursively binary partition the subjects according to the yes/no answer to the binary question. In order to decide which feature and which threshold to use, at each node, all features were tested with all possible threshold values (that is all median values between successive values of the considered feature). To identify the best feature/threshold combination, the Gini impurity can be computed to measure the inequality among values of frequency distributions. As a perfect subset only contains subjects from one group (either MCI/dementia or healthy), it will show high frequency disparities in the two groups. A small degree of Gini impurity indicates a good discrimination between subjects who remained healthy and subjects who developed MCI/dementia. Hence, at each node, the descriptor/threshold combination which led to the lowest Gini impurity degree was chosen. In this study, the final decision tree was optimized using a cross-validation protocol and manually edited as follows. Ten trees were constructed by 10-fold cross-validation. For each one, the construction was stopped when less than 6 observations were found in a leaf. The final retained tree was the one that used the same descriptor for most of the trees which constitute robust splits. The final corresponding threshold was assigned to the modal value. Decisions trees were constructed using library rpart in R 2.13.0 (http://www.r-project.org/).

#### Features of WML patterns and relationship with risk of MCI/dementia

The features of the participants belonging to the different WML patterns were compared with ad hoc statistical tests (polytomous regression for categorical variables and ANOVA with Tukey's HSD comparison for continuous variables). To examine the relationship between WML patterns and risk of MCI/dementia independently of potential confounders over the 7-year follow-up period, Cox proportional hazard regression models with delayed entry [31] were used with age as the basic timescale and birth as the time origin. Participants who died without MCI/dementia were censored at the age of death and also individuals who missed three consecutive follow-up visits after baseline. Analyses were repeated using successive adjustments with possible confounders. Results of proportional-hazard regression analyses were expressed as hazard ratios (HR) with 95% confidence intervals (CI). Cox proportional hazard regression models were performed using the SAS software version 9.1 (SAS Institute, Inc., Cary, NC).

## Results

### Participants' characteristics

**Figure 1:**
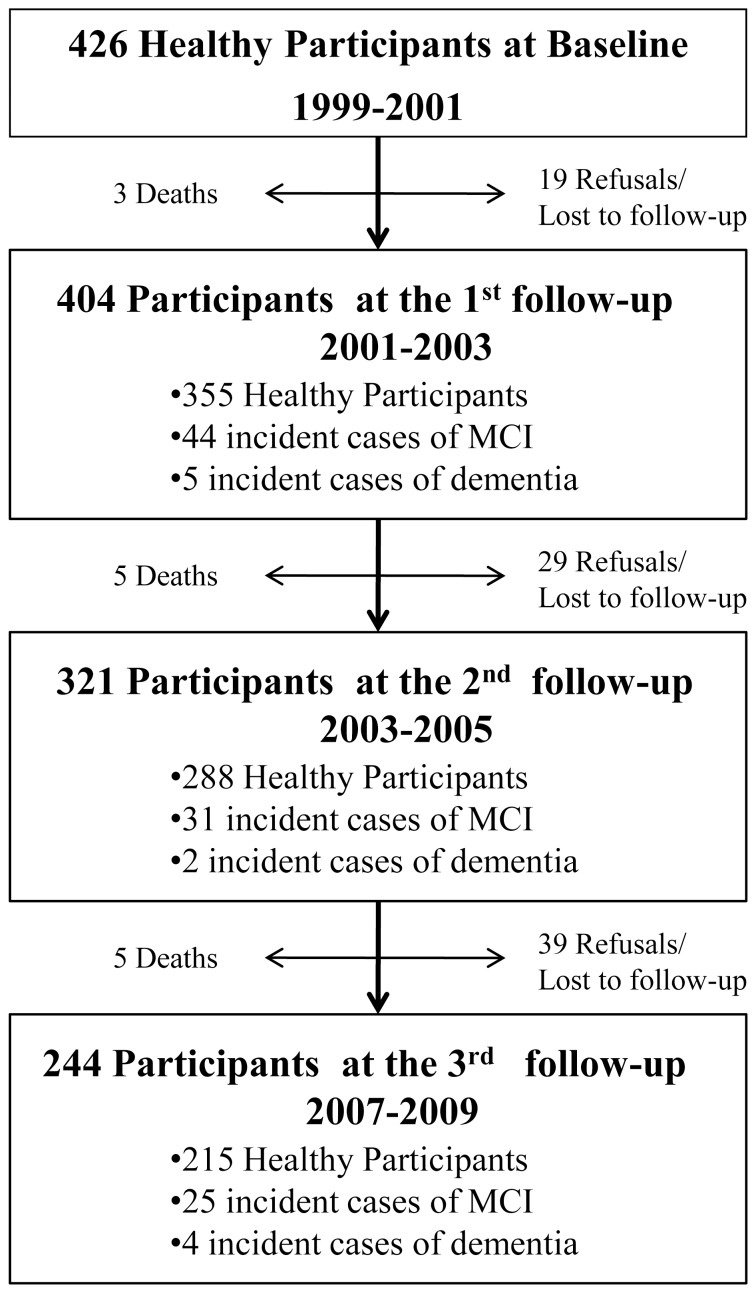
Flow Chart. Study design and flow of participants through the study.

Table 1 describes the characteristics of the 426 participants. Four hundred and four individuals (95%) had at least 1 follow-up examination (Figure 1). During 2637 person-years of follow-up (mean per person: 5.4 years), 11 participants developed dementia (incidence rate: 4.2/1000 person-years) and 100 MCI (incidence rate: 38/1000 person-years). Thirteen participants died (3%) and 87 (20%) were lost to follow-up or refused to continue the study. Subjects who died or were lost to follow-up were significantly older and had significantly lower baseline MMSE scores and significantly higher WML volume (data not shown). Conversely, they did not show differences in gender distribution.

**Table 1:** Characteristics of the study participants (n = 426).

	Mean (SD)
Age at baseline (years)	71.1 (4.0)
MMSE at baseline	27.5 (1.8)
Duration of follow-up (years)	5.4 (2.7)
Total brain volume (ml)	1017 (101)
Brain atrophy (%)	15.2 (2.4)
Hippocampal volume (ml)¶	5.8(0.8)
WML (ml)	
Total	1.78 (3.56)
Frontal region	1.15 (2.31)
Parietal region	0.56 (1.37)
Temporal region	0.05 (0.13)
Occipital region	0.03 (0.10)

	% (n)
Female gender	55 (233)
MCI/dementia (7 years of follow-up)	26 (111)
Presence of silent brain infarcts§	42 (174)
Education level	
No formal education	24 (102)
Primary school	28 (120)
Secondary education	20.5 (87)
Higher/university education	27.5 (117)

¶ n = 424.

§ n = 418.

### Decision trees and WML patterns

**Figure 2:**
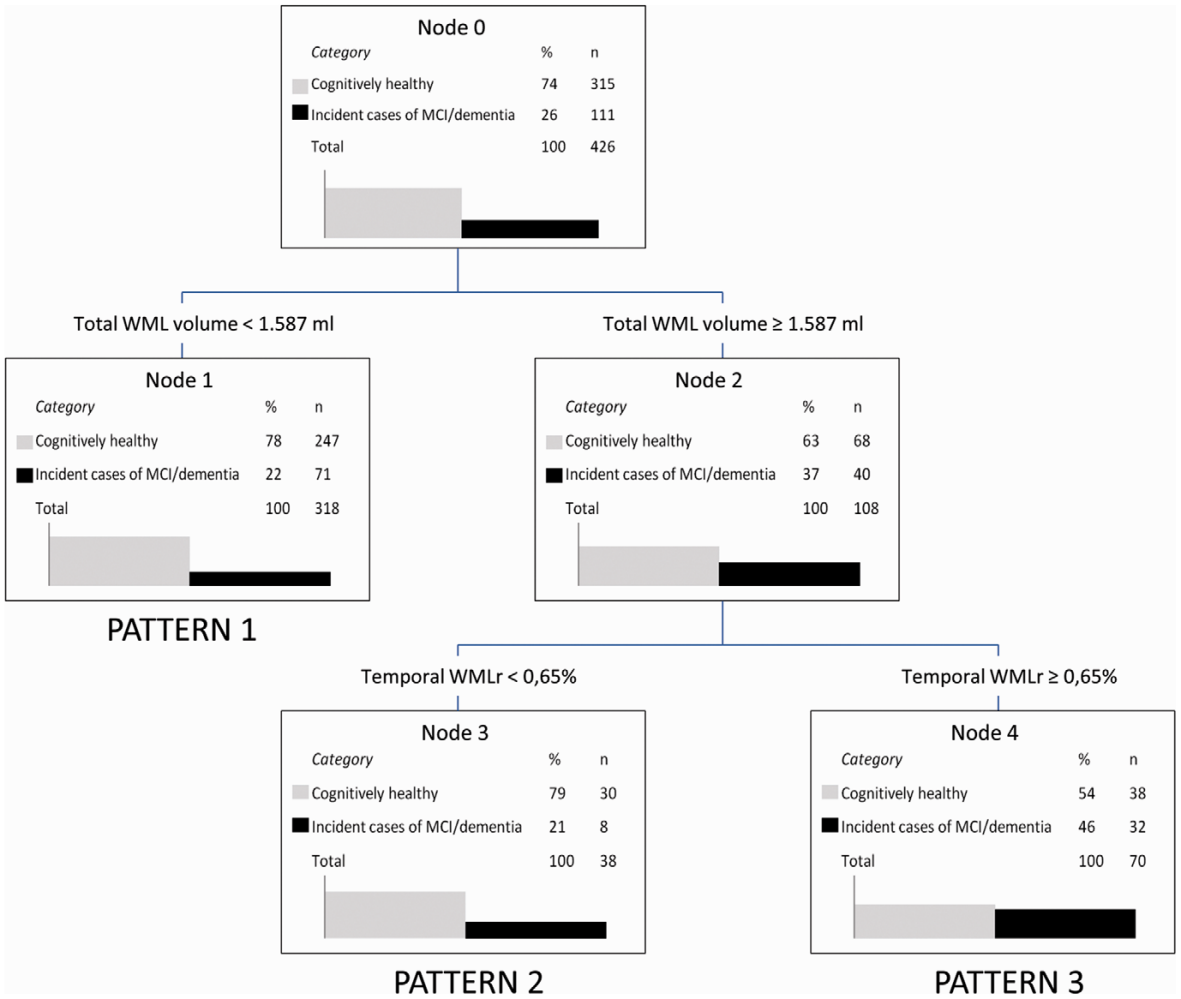
Decision tree (built using the data on total WML volume and on relative WML volume in the frontal, parietal, temporal and occipital regions as potential descriptors). The three distribution patterns could be used to discriminate between subjects who developed MCI/dementia and subjects who remained cognitively stable. Sensitivity: 29%, specificity: 88%, positive predictive value: 46%, negative predictive value: 78%. Temporal WMLr: (temporal WML volume/total WML volume) ×100.

The final decision tree revealed two main descriptors (total WML volume and temporal WMLr) and three distinct WML anatomical patterns that defined three groups of participants (Figure 2). Total WML volume (WML volume< or ≥1.587 ml) was the first descriptor and defined pattern 1 (total WML volume<1.587 ml). Among subjects with a total WML volume ≥1.587 ml, temporal WMLr was the second descriptor and defined pattern 2 (temporal WMLr<0.65%) and pattern 3 (temporal WMLr≥0.65%). We observed a poor correlation (Pearson correlation coefficient r = −0.05, p = 0.34) between total WML volume and temporal WMLr, which confirms that they provide low redundancy and complementarity in discriminating subjects who develop MCI/dementia from subjects who remain cognitively stable (29% sensitivity, 88% specificity, 46% positive predictive value, 78% negative predictive value).

The characteristics of three groups of participants defined by the three WML patterns are shown in Table 2. No significant differences were found in the level of education, history of vascular pathology, frequency of APOE ε4 carriers, MMSE score at baseline and hippocampal volume. However, the pattern 3 subjects were significantly older than those included in the pattern 1 group. No significant differences were found between pattern 2 and 3 groups concerning total brain volume, brain atrophy, total WML load, hypertension, history of stroke and presence of SBI. The pattern 1 group showed significantly lower total WML load, hypertension frequency and history of stroke than the pattern 2 and 3 groups. Subjects in the pattern 3 group had a significantly lower follow-up duration than those in the pattern 1 group. The pattern 2 group was characterized by higher frequency of depressive symptomatology. Overall the three groups could easily be differentiated based on WML distribution.

**Table 2:** Characteristics of the different WML distribution patterns.

	Pattern 1 n = 318	Pattern 2 n = 38	Pattern 3 n = 70
		Mean (SD)	
Age (years)	70.7(3.9)	71.6(4.0)	72.4(4.0)*
Duration of follow-up (years)	5.7(2.5)	5.0(2.8)	4.5(2.9)*
Total brain volume (ml)	1008(94)	1056(134)*	1037(103)
Brain atrophy (%)	14.9(2.2)	16.6(3.3)*	15.8(2.6)*
Hippocampal volume (ml)	5.8(0.7)	5.7(0.9)	5.8(0.8)
Total WML volume (ml)	0.45(0.40)	5.4(5.2)*	5.9(5.6)*
MMSE at baseline	27.5(1.7)	27.2(1.5)	27.3(2.1)

		% (n)	
Female	55(174)	42(16)	61(43)
MCI/dementia (7 years of follow-up)	22(71)	21(8)	46(32)* †
Education level			
No formal education	23(74)	29(11)	24(17)
Primary school	29(92)	31(12)	23(16)
Secondary education	21(65)	16(6)	23(16)
Higher/university education	27(87)	24(9)	30(21)
History of stroke	0.6(2)	5(2)*	6(4)*
Presence of silent brain infarcts§	32(102)	72(26)*	70(46)*
High blood pressure	39(123)	58(22)*	63(44)*
History of vascular pathology	5(16)	11(4)	7(5)
Depressive symptomatology	14(45)	32(12)*	9(6)†
APOE ε4 carriers	18(58)	21(8)	27(19)

The characteristics of the three WML patterns were compared using ad hoc statistical tests (polytomous regressions for categorical variables, and ANOVA with Tukey's HSD comparison for continuous variables).

* p value <0.05 versus Pattern 1.

† p value <0.05 versus Pattern2.

¶ n _pattern 1_ = 317, n _pattern 2_ = 37, n _pattern 3_ = 70.

§ n _pattern 1_ = 316, n _pattern 2_ = 36, n _pattern 3_ = 66.

#### Pattern 1 group

**Figure 3:**
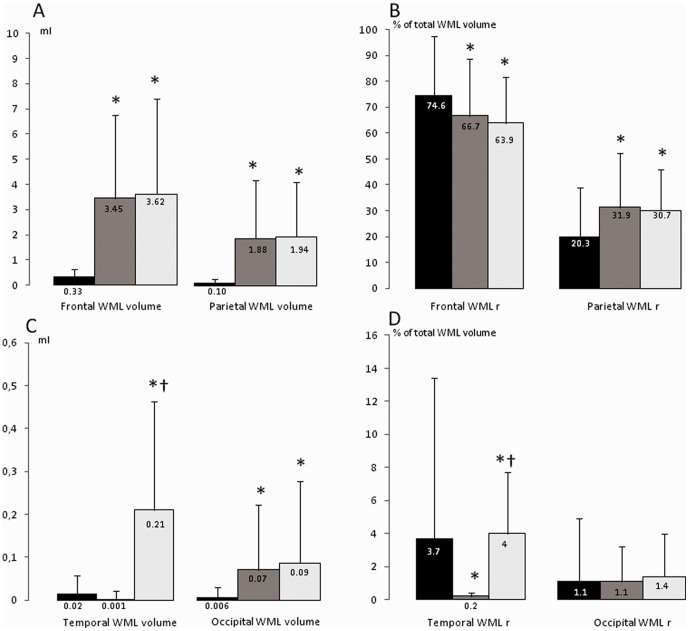
Mean absolute and relative regional WML volumes for the three patterns (black columns: pattern 1, grey columns: pattern 2, white columns: pattern 3). The scales of the graphics are different for the frontal/parietal and the temporal/occipital regions. (A) Frontal and parietal absolute WML volumes. (B) Relative (in percentage) WML volumes in the frontal (frontal WMLr) and parietal regions (parietal WMLr). (C) Temporal and occipital absolute WML volumes. (D) Relative (in percentage) WML in the temporal (temporal WMLr) and occipital regions (occipital WMLr). * p <0.01 versus pattern 1 (Tukey's HSD test).† p<0.01 versus pattern 2 (Tukey's HSD test).

Pattern 1 group was characterized by lower total (Table 2, Figure 2) and regional (frontal, parietal and occipital) WML volumes than the two other groups (p<0.01). The temporal WML volume was lower only in comparison to that of the pattern 3 group (p<0.01) (Figure 3). Comparison of the relative WML volumes in the different anatomical regions revealed that parietal WMLr was significantly lower and frontal WMLr was significantly higher in the pattern 1 group than in the two other groups. Temporal WMLr in the pattern 1 group was significantly higher than in pattern 2 (p<0.01), but significantly lower than in the pattern 3 group. Occipital WMLr was similar in the three groups (Figure 3).

#### Pattern 2 group

The main features of the pattern 2 group were high total and regional WML volumes in comparison to the pattern 1 group (Table 2, Figure 2) and low absolute and relative temporal WML volumes in comparison to both pattern 1 and pattern 3 groups (Figure 3).

#### Pattern 3 group

Like the pattern 2 group, pattern 3 subjects were characterized by high total and regional WML volumes (Table 2, Figure 2 and 3). However, pattern 3 absolute and relative temporal WML volumes were significantly higher than in the other two groups (Figure 3).

### WML patterns and risk of MCI/dementia

Cox proportional hazard regression models were used to examine the relationship between the three WML patterns and the risk of MCI/dementia. Potential confounders (age, gender, education level, hypertension, history of vascular pathology, APOE 4 genotype, depressive symptomatology, total brain volume, brain atrophy, hippocampal volume and presence of SBI) that were not taken into account in the decision tree were entered successively in the different models (Table 3).

**Table 3:** Cox proportional-hazard regression models for transition to MCI/dementia during the 7-year follow-up (n = 426, n. of events = 111).

			Groups			
	Pattern 2 versus pattern 1		Pattern 3 versus pattern 1		Pattern 3 versus pattern 2	
Models	HR (95% CI)	*p*	HR (95% CI)	*p*	HR (95% CI)	*p*
Model 1: Age*, Sex, Education level	1.04(0.50–2.18)	0.91	2.60(1.69–3.99)	<0.01	2.49(1.13–5.47)	0.02
Model 2: model 1+History of vascular pathology, Hypertension	1.04(0.49–2.17)	0.93	2.58(1.66–4.00)	<0.01	2.49(1.13–5.46)	0.02
Model 3: model 2+APOE 4 genotype, Depressive symptomatology	0.83(0.38–1.78)	0.62	2.65(1.70–4.13)	<0.01	3.20(1.42–7.24)	<0.01
Model 4: model 3+Total brain volume	0.79(0.36–1.70)	0.54	2.55(1.63–3.99)	<0.01	3.24(1.44–7.33)	<0.01
Model 5†: model 4+Hippocampal volume, Brain atrophy, Presence of silent brain infarcts	0.63(0.28–1.42)	0.26	2.28(1.41–3.67)	<0.01	3.63(1.54–8.54)	<0.01

* Cox proportional hazard regression models with delayed entry were performed with age as the basic timescale and birth as the time origin.

† In model 5, n = 416 and n. of events = 108.

Diagnosis of MCI/dementia during the 7 years of follow-up was significantly associated with pattern 3 in all models. In the model adjusted for all potential confounders (model 5 in Table 3), the MCI/dementia HR for the pattern 3 group was 2.28 (1.41–3.67) when compared to the pattern 1 (p<0.01) and 3.63 (1.54–8.54) when compared to the pattern 2 group (p<0.01). Conversely, the pattern 2 group did not show increased risk of MCI/dementia in comparison to the pattern 1 group in any of the used models.

## Discussion

In this study, we examined whether a specific WML profile (density and location) could be associated with an increased risk of MCI/dementia. In our study population, we found that people with high total WML load and high percentage of WML in the temporal lobe (≥0.65%) were more at risk of MCI/dementia over a 7-year period, independently of potential confounders and other structural brain changes detected by MRI.

Previous studies compared WML distribution in healthy and dementia groups that were determined using clinical criteria which are the results of heterogeneous pathologic processes (top-down approach). In this analysis, we employed an original bottom-up approach in which all participants were initially included in one single group that comprised all cognitive statuses and then the regional and total WML volumes were the only components used in a decision tree to specifically discriminate between healthy and cognitively impaired subjects. Our approach focused on the specific relationship between WML distribution and risk of MCI/dementia, independently of the influence of other known contributors to cognitive impairment. The groups provided by the optimized decision tree therefore did not perfectly discriminate between healthy and MCI/dementia subjects. This relative low sensitivity is due to the fact that WML alone cannot predict the risk of MCI/dementia. However the robust specificity and negative predictive values indicate that WML distribution can be used to differentiate people who are unlikely to decline cognitively. We then verified that these findings were independent of potential confounders, which were not considered in the decision tree, by multivariate analysis (Cox proportional-hazard regression models).

The optimized decision tree revealed three separate WML patterns. Although the WML load was more severe in the frontal region in all patterns, the relative frontal volume (WMLr volume) was significantly lower in pattern 2 and 3 than in pattern 1. Conversely, the total and the parietal and occipital WML volumes were higher in pattern 2 and 3 than in pattern 1. Although MRI data were collected only at baseline, these results suggest a spatial progression of WML and are in agreement with previous findings about the antero-posterior progression of WM deterioration [8], [9].

The decision tree identified two different patterns (2 and 3) in participants with severe WML load, but only pattern 3 was significantly associated with higher risk of transition to MCI/dementia, suggesting that severe WML load is not sufficient to explain this association. Although the total WML load was similar in patterns 2 and 3, pattern 3 was characterized by a significantly higher temporal WML load (both absolute and as a percentage of the total volume) than pattern 2. As age and hypertension were not significantly different in these two groups, they cannot explain this location difference. On the other hand, the temporal region is the first brain area to be affected by AD pathology [32]. Further research is needed to investigate whether, in addition to its ischemic origin, a severe WML load in the temporal region could be related to the grey matter pathology observed specifically in the temporal region in AD [14], [33].

Our findings suggest that beyond a given level of WML load, a specific WML distribution is observed with an increased proportion of WML in the temporal region. This feature is in turn associated with increased risk of progression towards MCI/dementia. This result is in agreement with previous reports indicating that WM deterioration in the temporal region is the most consistent finding in patients with AD [33], [34], [35], [36]. In addition, our study shows a disease-specific WML localization (pattern 3) that is independent of the global WM changes. Pattern 3 (associated with higher risk of transition to MCI/dementia) was observed before the onset of symptoms, because all subjects were considered cognitively normal when MRI was carried out. These findings may thus be more useful for the detection of patients in the early prodrome of dementia than cognitive testing, and also for selecting elderly people at increased risk for clinical trials.

Pattern 2 was characterized by higher frequency of depressive symptomatology. The association between cerebrovascular disease and depressive symptoms is well-known [37]. However, further investigations are needed to determine whether there is a WML distribution pattern that may be specifically associated with depressive symptomatology independently of multiple confounding factors.

Some limitations of our study should be considered. Our study sample shows a relative low incidence of dementia (4.2 per 1000 person-year) [38], certainly because it included only participants aged 80 years and younger. However, despite the low number of dementia cases, our results are consistent with previous studies. Moreover, we mapped the WML load in the different brain lobes, while some other studies distinguished periventricular from deep WML. However, the different impact of these WML locations upon cognitive function is controversial [39]. Finally, our method quantified WML load per lobe but did not determine which particular WM structure in the temporal region was involved. The strengths of our study include the large number of available MRI scans and the length of the follow-up period (7 years).

## Conclusion

Our MRI data suggest that WML load shows an antero-posterior gradient in all subjects. However, above a certain threshold of WM damage, WML load in the temporal region is significantly higher in some subjects, who are more likely to develop MCI or dementia. The association between WML and risk of MCI/dementia therefore depends on both the extent and location of WML. As this particular distribution of lesions is observed before the onset of clinical symptoms, it could facilitate the selection of patients at risk of prodromal dementia.
